# Effects of vitamin D supplementation during pregnancy on bone health and offspring growth: A systematic review and meta-analysis of randomized controlled trials

**DOI:** 10.1371/journal.pone.0276016

**Published:** 2022-10-13

**Authors:** Ting Luo, Yunzhu Lin, Jiayue Lu, Xianghong Lian, Yuanchao Guo, Lu Han, Yixin Guo

**Affiliations:** 1 Department of Pharmacy, West China Second University Hospital, Sichuan University, Chengdu, People’s Republic of China; 2 Evidence-Based Pharmacy Center, West China Second University Hospital, Sichuan University, Chengdu, People’s Republic of China; 3 Key Laboratory of Birth Defects and Related Diseases of Women and Children, Ministry of Education, Sichuan University, Chengdu, People’s Republic of China; Nanjing Medical University, CHINA

## Abstract

**Background:**

Whether vitamin D supplementation during pregnancy is beneficial to bone health and offspring growth remains controversial. Moreover, there is no universal agreement regarding the appropriate dose and the time of commencement of vitamin D supplementation during pregnancy.

**Objective:**

We aimed to systematically review the effects of vitamin D supplementation during pregnancy on bone development and offspring growth.

**Methods:**

A literature search for randomized controlled trials (RCTs) was performed in 7 electronic databases to identify relevant studies about the effects of vitamin D supplementation during pregnancy on bone development and offspring growth from inception to May 22, 2022. A Cochrane Risk Assessment Tool was used for quality assessment. Vitamin D supplementation was compared with placebo or standard supplements. The effects are presented as the mean differences (MDs) with 95% CIs. The outcomes include bone mineral content (BMC), bone mineral density (BMD), bone area (BA), femur length (FL) and humeral length (HL); measurement indicators of growth, including length, weight and head circumference; and secondary outcome measures, including biochemical indicators of bone health, such as the serum 25(OH)D concentration. Additionally, subgroup analyses were carried out to evaluate the impact of different doses and different initiation times of supplementation with vitamin D.

**Results:**

Twenty-three studies with 5390 participants met our inclusion criteria. Vitamin D supplementation during pregnancy was associated with increased humeral length (HL) (MD 0.13, 95% CI 0.06, 0.21, I^2^ = 0, P = 0.0007) during the fetal period (third trimester). Vitamin D supplementation during pregnancy was associated with a significantly increased length at birth (MD 0.14, 95% CI 0.04, 0.24, I^2^ = 24%, P = 0.005) and was associated with a higher cord blood 25(OH)D concentration (MD 48.74, 95% CI 8.47, 89.01, I^2^ = 100%, P = 0.02). Additionally, subgroup analysis revealed that birth length was significantly higher in the vitamin D intervention groups of ≤1000 IU/day and ≥4001 IU/day compared with the control group. Prenatal (third trimester) vitamin D supplementation was associated with a significant increase in birth length, while prenatal (second trimester) vitamin D supplementation was associated with a significant increase in birth weight.

**Conclusion:**

Vitamin D supplementation during pregnancy may be associated with increased humeral length (HL) in the uterus, increased body length at birth and higher cord blood 25(OH)D concentration. Evidence of its effect on long-term growth in children is lacking. Additional rigorous high-quality, long-term and larger randomized trials are required to more fully investigate the effects of vitamin D supplementation during pregnancy.

## 1. Introduction

Women undergo a number of physiological changes during pregnancy, such as weight gain, hormonal changes, cardiac and hematological alterations, oxygen demand, etc. Nutritional requirements during pregnancy differ considerably from those of nonpregnant populations [1]. A pregnant woman’s nutritional status during pregnancy is critical both for her health and for her offspring’s health. Pregnant women require a healthy diet that includes an adequate intake of energy, protein, vitamins and minerals to meet the increased maternal and fetal needs [2]. In recent years, increasing attention has been given to nutritional supplements for pregnant women. Vitamin D has attracted much attention due to the high global prevalence of vitamin D deficiency.[2–48]

Vitamin D is a fat-soluble vitamin, that is important for maintaining normal levels of calcium and phosphate in the blood [2] and it has essential calcium absorption, metabolism and bone health functions and atypical effects that may affect various aspects of health [3]. The importance of vitamin D for the promotion and maintenance of bone health throughout the life cycle has been well established; vitamin D is an essential element for the bone mineralization that begins in utero and continues throughout childhood until early adulthood [4], and a low bone mineral content (BMC) and low bone mineral density (BMD) contribute to fracture risk in childhood and osteoporotic fractures in later life [5, 6]. Vitamin D deficiency during pregnancy is prevalent worldwide, especially in developing countries [7–9].

Low vitamin D level status during pregnancy may expose the offspring to a suboptimal nutritional environment during critical phases of fetal development and may have long-term effects on offspring health outcomes [3]. In several observational studies, maternal vitamin D status has been associated with lower bone mass in offspring [10–13]. However, evidence that maternal vitamin D repletion during pregnancy improves offspring bone mass is lacking. It is unclear whether vitamin D supplementation during pregnancy will be beneficial to bone health and offspring growth. Moreover, there is no universal agreement regarding the appropriate dose and the time of commencement of vitamin D supplementation during pregnancy. We conducted a systematic review and meta-analysis of randomized controlled trials (RCTs) with the aim of evaluating the effects of vitamin D supplementation during pregnancy on bone health and offspring growth. We compared the effects of different doses and different initiation times of supplementation to guide future vitamin D supplementation strategies.

## 2. Materials and methods

This meta-analysis was performed according to the recommendations in the Preferred Reporting Items for Systematic Reviews and Meta Analyses (PRISMA) statement and the guidelines described in the Cochrane Handbook [14].

### 2.1 Search strategy

Electronic literature retrieval was performed on 3 English electronic databases (PubMed, Embase, Cochrane Library) and 4 Chinese electronic databases (China National Knowledge Infrastructure, Wan Fang Database, Chinese Biomedical Literature Database, VIP Database for Chinese Technical Periodicals). The final literature search was performed on May 22, 2022. Randomized controlled trials on the effects of vitamin D supplementation during pregnancy on bone health and offspring growth were collected. The systematic literature search was based on the following retrieval strategies: controlled vocabulary (i.e., maternal, prenatal, cord, in utero, pregnancy, mother, gestation, antenatal, perinatal, vitamin D, Vit D, 25(OH)D, 25OH-vitamin D, 25OHD, 25-hydroxyvitamin D, offspring, infant, perinatal, neonatal, early life, child*, adolescen*, adult*, fetus) were included and systematically combined (AND/OR). References cited in these articles were manually searched to identify additional RCTs.

### 2.2 Inclusion and exclusion criteria

The studies had to fulfill the following criteria to be eligible for inclusion: (a) Participants: the offspring of women who received vitamin D supplements during pregnancy were included. (b) Interventions and comparisons: pregnant women were given vitamin D supplementation vs. placebo or standard supplement, or, in cases of co-intervention, continuous additional supplements across treatment groups. (c) Outcome measurements: the main outcome measures including bone mineral content (BMC), bone mineral density (BMD), bone area (BA), femur length (FL) and humeral length (HL); measurement indicators of growth including length, weight and head circumference; and secondary outcome measures including biochemical indicators of bone health such as serum 25(OH)D concentration. (d) Type of study: randomized controlled trial (RCT). (e) Restricted to published in Chinese or English. The exclusion criteria were as follows: pregnant women with pregnancy complications or chronic metabolic diseases; data from case reports, reviews, or animal studies; no outcome of interest, and duplicate published studies.

### 2.3 Data extraction

Two reviewers independently extracted the data based on a previously designed data extraction table. Data were extracted, including the author, year of publication, country, experimental design, sample size, intervention measure, dose, initiation and duration of supplementation, and any outcome that met the inclusion criteria. The data describing the same outcomes were converted to the same units. Two independent reviewers screened the titles and abstracts to identify potentially eligible articles. They independently applied the eligibility criteria to perform the final selection. When the reviewers disagreed about an article, they discussed it and came to a consensus. If no agreement could be reached, a third reviewer made the final decision.

### 2.4 Quality assessment

Two reviewers independently evaluated the methodological quality of each included study by applying the Cochrane risk of bias tool of RCTs.

### 2.5 Statistical analysis

The statistical analysis was carried out using Review Manager, version 5.3. For continuous data, we calculated the sample size–weighted mean difference (MD) when outcomes were measured in the same way by multiple studies. We used forest plots to show the point estimate (95% CIs) for each study. The I2 statistic was used to quantify the degree of heterogeneity across studies. If I2≥50%, heterogeneity was considered significant, and we pooled the results using a random-effects model. Otherwise, a fixed-effects model was applied. A p value less than 0.05 was considered significant for our systematic review. Descriptive analysis was carried out for studies that could not be statistically analyzed. Subgroup analyses were carried out to evaluate the impact of different doses and different initiation times of vitamin D supplementation strategies. Additionally, a funnel plot was used to evaluate publication bias. Sensitivity analysis was performed to assess the potential influences of a single study on the pooled effect size. It was conducted by omitting single studies one at a time for each meta-analysis to screen for significant alterations of the pooled effect size.

## 3. Results

### 3.1 Study selection

**Fig 1:**
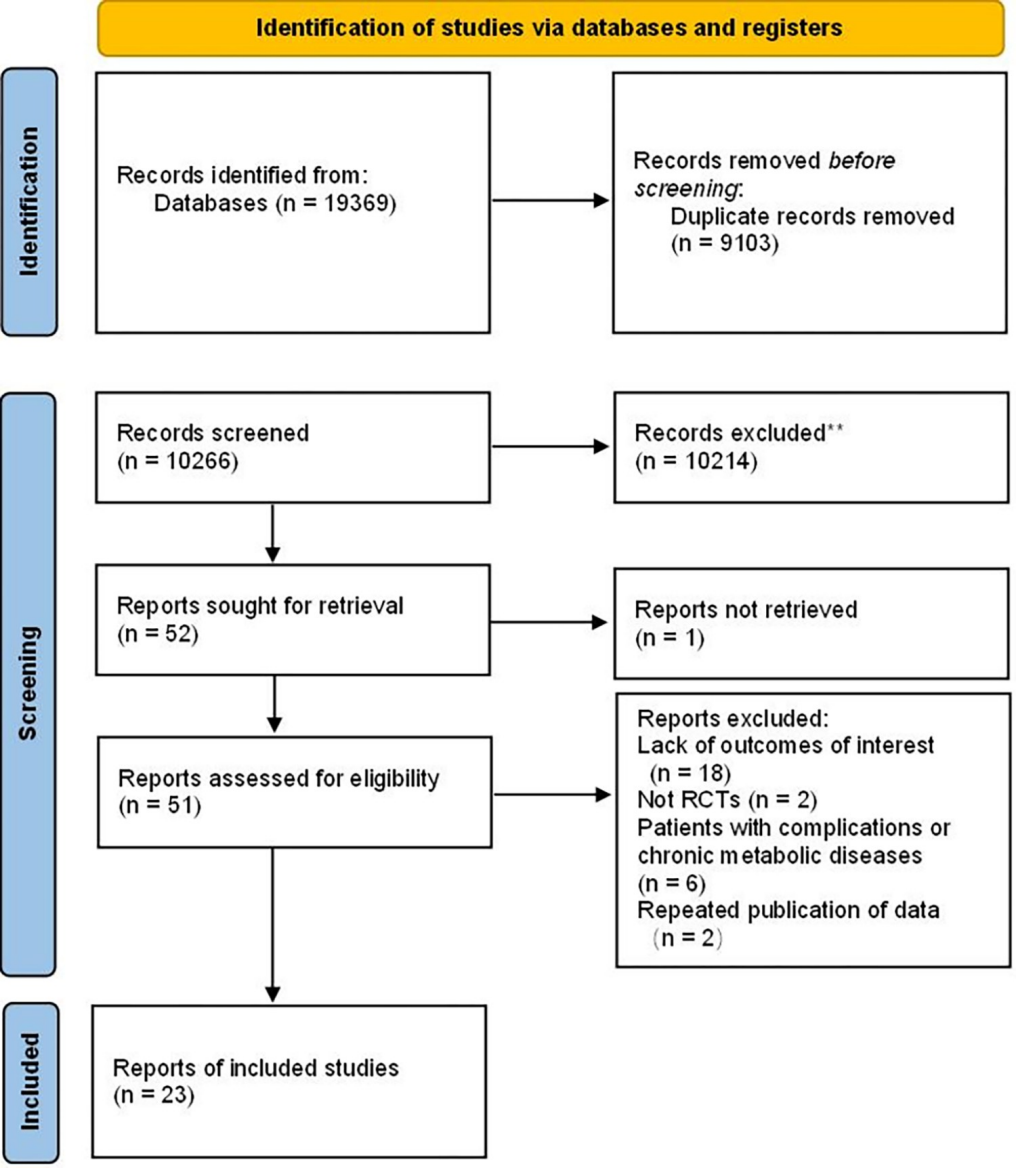
Flow chart of literature screening and the selection process.

A total of 19369 articles were identified for preliminary screening. After screening the titles and abstracts, we read 48 full-text articles, 23 of which were included [15–37] in this systematic review (Fig 1).

### 3.2 Basic characteristics of the included trials

This systematic review included 23 randomized controlled trials with a total of 5390 participants. Thirteen studies [17, 18, 22–24, 26, 27, 30–32, 34, 35, 37] were placebo-controlled; nine studies [15, 16, 19–21, 25, 29, 33, 36] compared high and low doses (≤400 IU/day) of vitamin D; and one study [28] involved control groups without supplements. Fifteen studies [15, 16, 18, 19, 21–23, 25, 30–36] were supplemented with cholecalciferol. One study [37] was supplemented with ergocalciferol. Seven studies [17, 20, 24, 26–29] did not report the supplementary compound form. Three studies [19, 20, 30] conducted vitamin D and calcium supplementation in the treatment groups, but the vitamin D doses were different between the intervention groups and the control group. The characteristics of the included studies are detailed in Table 1.

**Table 1:** Characteristics of the included studies.

Source	Country	Sample size	Participants	Interventions	Initiation and Duration of Supplementation	Outcomes
Brustad 2020 [15]	Danish	294 vs 286	22–26 weeks of gestation	cholecalciferol, 2800 IU/day vs 400 IU/day	from 24 weeks of gestation until 1 week after birth	TBLH-BMD, WB-BMD and head-BMD at 3 and 6 years, TBLH-BMC, WB-BMC and head-BMC at 3 and 6 years, Head circumference at 6 years
Chen 2020 [16]	United States	196 vs 191	10–14 weeks of gestation	cholecalciferol, 4400 IU/day vs 400 IU/day	from 10–14 weeks until delivery	Birth head circumference, Birth weight, Cord blood 25(OH)D concentration
Vafaei 2019 [17]	Iran	68 vs 62	20–35-years-old healthy primigravida pregnant woman	Vitamin D, 1000 IU/day vs placebo	from two weeks after menstrual retardation until the last sonography at 34 weeks of gestational age	FL, HL
Roth 2018 [18]	Bangladesh	779 vs 259	17and 24 weeks of gestation	cholecalciferol, 4200~28000 IU/week vs placebo	from 17 to 24 weeks of gestation until birth	Birth head circumference, Birth weight, Birth length, Length at 1 year
Sahoo 2017 [19]	India	36 vs 16	age over 18 years, singleton pregnancy, 14–20 weeks of geatation	cholecalciferol, 60000IU every 4 or 8 weeks vs 400IU/day	from 14–20 weeks until delivery	Infant birth Calcium concentration, WB-BMD and WB-BMC at 12–16 months, Birth weight, Birth length, Cord blood 25(OH)D concentration
Abotorabi 2017 [20]	Iran	55 vs 55	22–26 weeks of gestation	Vitamin D, 5000U/week and 400U/day vs 400U/day	for 8 weeks until delivery	Birth head circumference, Birth weight, Birth length
Thiele 2017 [21]	United States	8 vs 8	age over 18 years, 24–28 weeks of gestation	cholecalciferol, 3800IU/day vs 400IU/day	from enrollment until 4 to 6 weeks postpartum	Birth head circumference, Birth weight, Birth length
Cooper 2016 [22]	UK	479 vs 486	age over 18 years, singleton pregnancy, less than 17 weeks of gestation	cholecalciferol, 1000IU/day vs placebo	from 14 weeks of gestation until delivery	WB-BMD, WB-BMC and WB-BA of the neonate, Birth head circumference, Birth weight, Birth length
Vaziri 2016 [23]	Iran	62 vs 65	aged 18 years or older, singleton pregnancy, 26–28 weeks of gestation	cholecalciferol, 2000IU/day vs placebo	from 26–28 weeks of gestation until delivery	WB-BMD, WB-BMC and WB-BA of the neonate, Head circumference at birth, 4 weeks and 8 weeks of postpartum, Weight at birth, 4 weeks and 8 weeks of postpartum, Length at birth, 4 weeks and 8 weeks of postpartum
Naghshineh 2016 [24]	Iran	68 vs 70	less than 16 weeks of gestation	Vitamin D, 600 IU/day vs placebo	from 16 weeks of gestation until delivery	Birth weight
Zerofsky 2016 [25]	United States	25 vs 26	aged over 18 years, singleton pregnancy, <20 weeks of gestation	cholecalciferol, 2000 IU/day vs 400 IU/day	from 20 weeks of gestation until delivery	Birth weight
Khan 2016 [26]	Pakistan	36 vs 49	12–16 weeks of gestation	Vitamin D, 4000 IU/day vs placebo	from 12–16 weeks of gestation until delivery	Birth weight
Charandabi 2015 [27]	Iran	42 vs 42	age 18 to 39 years, 25 to 30 weeks of gestation	Vitamin D, 1000 IU/day vs placebo	60 days	Birth head circumference, Birth weight, Birth length
Sablok 2015 [28]	Indian	108 vs 57	14–20 weeks of gestation, Primigravidae with singleton pregnancy	Vitamin D, one dose of 60000 IU or two doses of 120 000 IU or four doses of 120 000 IU vs no intervention	20, 24, 28 and 32 weeks	Birth weight
Mojibian 2015 [29]	Iran	186 vs 203	12–16 weeks of gestation	Vitamin D, 50000 IU every 2 weeks vs 400 IU/day	from 12 weeks of gestation until delivery	Birth head circumference, Birth weight, Birth length, Cord blood 25(OH)D concentration
Diogenes 2015 [30]	Brazilian	30 vs 26	age 13–19 years, Primigravidae with singleton pregnancy, 21–29 weeks of gestation	Cholecalciferol, 200 IU/day vs placebo	from 26 weeks of gestation until delivery	TBLH-BMD, TBLH-BMC and TBLH-BA at 5 weeks, FL, HL, Birth head circumference, Weight at birth and 5 weeks of postpartum, Length at birth and 5 weeks of postpartum
Karamali 2015 [31]	Iran	30 vs 30	aged 18–40 years, 22–26 weeks of gestation	cholecalciferol, 50000 IU every 14 days vs placebo	from 20 to 32 weeks of gestation	Birth head circumference, Birth weight, Birth length
Hossain 2014 [32]	Pakistani	86 vs 89	20 weeks of gestation, singleton pregnancy	cholecalciferol, 4000IU/day vs placebo	from 20 weeks until delivery	Birth head circumference, Birth weight, Birth length
Hashemipour 2014 [33]	Iran	55 vs 54	24–26 weeks of gestation, singleton pregnancy	cholecalciferol, 50000U each week for 8 weeks and 400U/day vs 400U/day	from 26 to 28 weeks of pregnancy until delivery	Birth head circumference, Birth weight, Birth length, Cord blood 25(OH)D concentration
Daniel 2013 [34]	Bangladesh	72 vs 75	18~35years, 26~30 weeks of gestation	cholecalciferol, 35000 IU/week vs placebo	from 26~30 weeks of gestation until delivery	FL, Head circumference at birth and 1 year, Weight at birth and 1 year, Length at birth and 1 year, Cord blood 25(OH)D concentration
Sabet 2012 [35]	Iran	25 vs 25	27 to 28 weeks of gestation	cholecalciferol, 10000 IU/month vs placebo	until term	Birth weight, Cord blood 25(OH)D concentration
Hollis 2011 [36]	United States	239 vs 111	12~16 weeks of gestation, singleton pregnancy	cholecalciferol, 2000-4000IU/day vs 400 IU/day	until delivery	Birth weight, Cord blood 25(OH)D concentration
Brooke 1980 [37]	England(Asian community)	59 vs 67	28–32 weeks of gestation	ergocalciferol, 1000IU/day vs placebo	until delivery	Birth head circumference, Birth weight, Birth length, Cord blood 25(OH)D concentration

**Abbreviations:** TBLH-BMD, total body less head bone mineral density; TBLH-BMC, total body less head bone mineral content; TBLH-BA, total body less head bone area; WB-BMD, whole body bone mineral density; WB -BMC, whole body bone mineral content; WB -BA, whole body bone area; FL, femur length; HL, Humeral length; 25(OH)D, 25 hydroxyvitamin D.

### 3.3 Risk of bias of the included clinical trials

**Fig 2:**
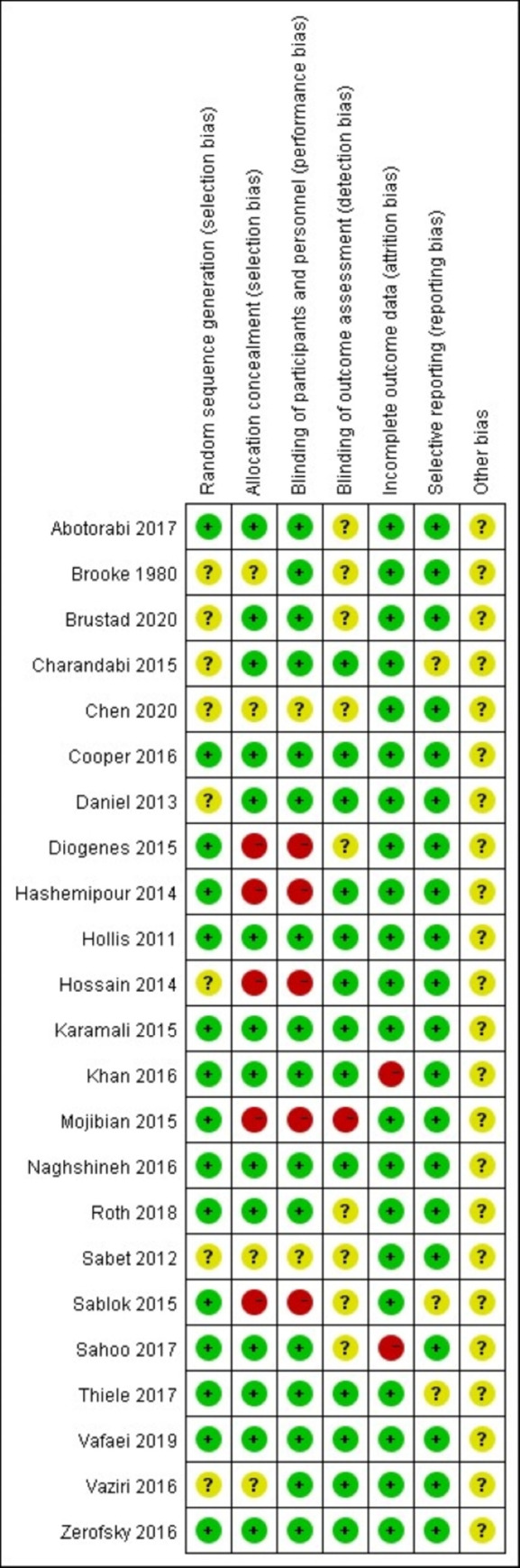
Risk of bias assessment of included studies. Notes: Green + dot, low risk of bias; yellow? dot, unclear risk of bias; red—dot, high risk of bias.

According to the Cochrane risk of bias tool, 7 aspects were evaluated. In terms of random sequence generation, 15 studies with a low risk of bias used sufficient random sequence generation methods, such as using a random number table or a computer-generated random number table, and 8 studies had an unclear risk of bias due to not reporting their randomization method. In terms of allocation concealment, 14 studies had a low risk of bias, 5 had a high risk of bias and 4 had an unclear risk of bias. For the blinding of participants and personnel, 16 studies had a low risk of bias, 5 had a high risk of bias and 2 had an unclear risk of bias. For a blinded method of results evaluation, 13 studies had a low risk of bias, 1 had a high risk of bias and 9 had an unclear risk of bias. For incomplete outcome data, there were 21 studies with a low risk of bias and 2 studies with a high risk of bias. In terms of selective reporting, there were 20 studies with a low risk of bias and 3 studies with an unclear risk of bias. Other biases were unclear in the included studies due to a lack of reporting (Fig 2).

### 3.4 Bone development assessment

#### 3.4.1 Bone mineral content (BMC)

A total of 1780 participants were examined in 5 RCTs [15, 19, 22, 23, 30], among which 3 studies [19, 22, 23] reported WB-BMC, 1 study [30] reported TBLH-BMC, 1 study [15] reported WB-BMC, TBLH-BMC and head-BMC. Dual-energy X-ray absorptiometry (DXA) was used in all of the studies to assess bone parameters. Offspring were assessed for BMC shortly after birth, and then at 5 weeks, between 12–16 months, 3 years, and 6 years of age.

**Fig 3:**
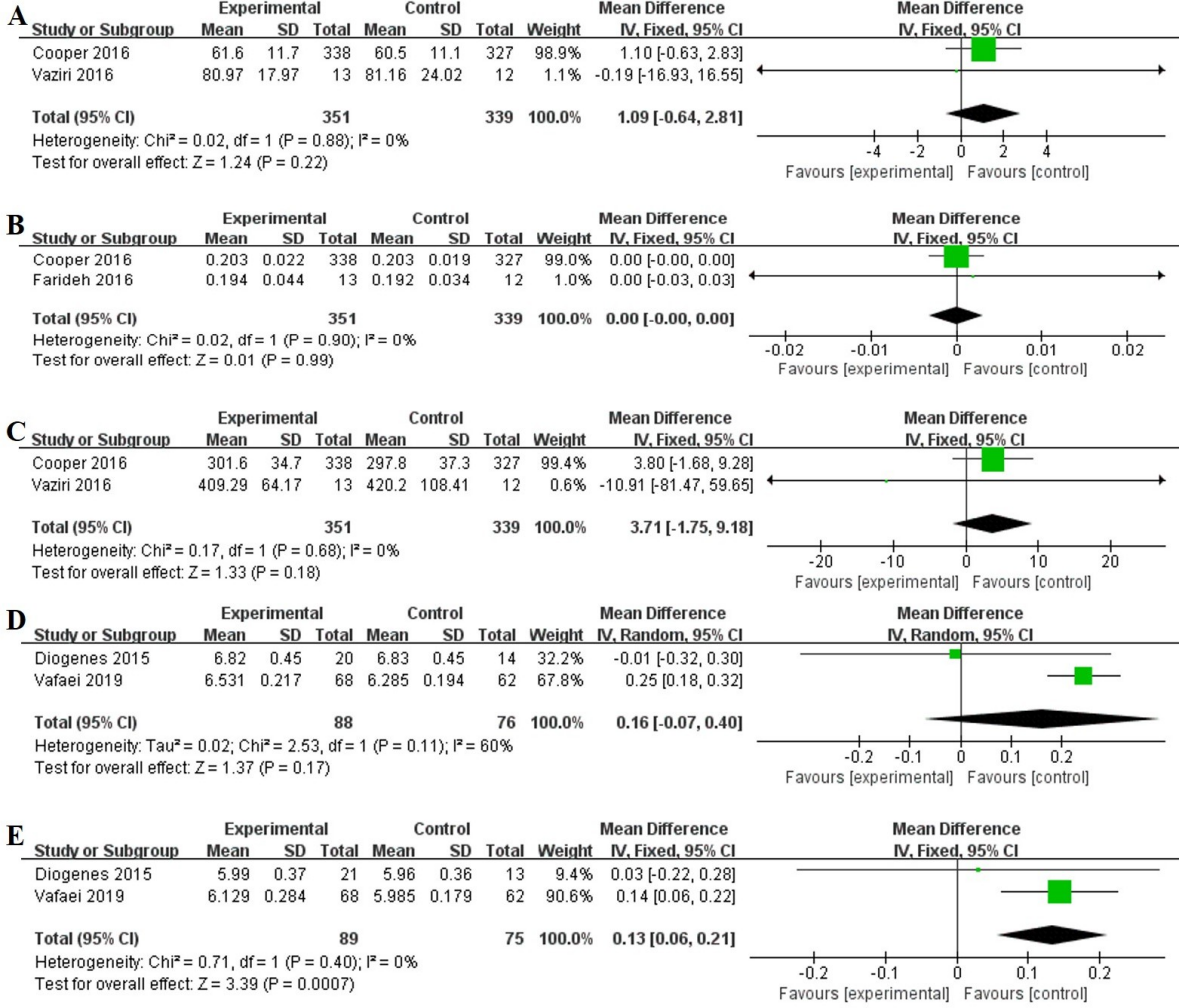
Forest plots of summary crude risk ratios of the association between vitamin D supplementation during pregnancy on bone development assessment of offspring. Notes: A, WB-BMC in neonates; B, WB-BMD in neonates; C, WB-BA in neonates; D, FL in the offspring during the fetal period (third trimester); E,HL in the offspring during the fetal period (third trimester).

Meta-analysis was performed on the only 2 RCTs measuring neonatal WB-BMC. There was no association between vitamin D supplementation during pregnancy and WB-BMC in neonates (MD 1.09, 95% CI -0.64, 2.81, I^2^ = 0%, P = 0.22) (Fig 3A). Brustad et al. [15] observed that high doses of vitamin D supplements during pregnancy can lead to higher WB-BMC and TBLH-BMC in the offspring in the first 6 years of life compared with the standard dose and it had the largest effect on bone mineralization outcomes when the child was born during winter or spring. Sahoo et al. [19] showed that vitamin D supplementation in pregnant women led to a higher WB-BMC in offspring at 12–16 months. Diogenes et al. [30] showed that calcium and vitamin D supplementation during pregnancy was not associated with TBLH-BMC at 5 weeks postpartum.

#### 3.4.2 Bone mineral density (BMD)

BMD was examined in 5 RCTs [15, 19, 22, 23, 30] involving 1780 participants, among which 3 studies [15, 19, 23] reported WB-BMD, 1 study [30] reported TBLH-BMD, and 1 study [15] reported WB-BMC, TBLH-BMC and head-BMC. Dual-energy X-ray absorptiometry (DXA) was used in all of these studies to assess bone parameters. BMD assessment was performed on the offspring as neonate and then at 5 weeks, between 12 and 16 months, 3 years and 6 years of age.

A meta-analysis was performed on the only 2 randomized controlled trials that measured neonatal WB-BMD. There was no association between vitamin D supplementation during pregnancy and neonatal WB-BMD (MD 0.00, 95% CI -0.00, 0.00, I^2^ = 0%, P = 0.99) (Fig 3B). Brustad et al. [15] observed that high doses of vitamin D supplements during pregnancy, compared with standard doses, resulted in higher head BMD in the offspring at age 6. Sahoo et al. [19] showed that vitamin D supplementation of pregnant women led to a higher WB-BMD in offspring at 12–16 months. Diogenes et al. [30] showed that calcium and vitamin supplementation during pregnancy was not associated with TBLH-BMD at 5 weeks postpartum.

#### 3.4.3 Bone area (BA)

BA was examined in 3 RCTs [22, 23, 30] involving 1148 participants, among which 2 studies [22, 23] reported WB-BA, and 1 study [30] reported TBLH-BA. Dual-energy X-ray absorptiometry (DXA) was used in all of these studies to assess bone parameters. BA assessment was performed in the offspring at birth and at 5weeks.

A meta-analysis was performed on the only 2 randomized controlled trials measuring neonatal WB-BA. There was no association between vitamin D supplementation during pregnancy and WB-BA in neonates (MD 3.71, 95% CI -1.75, 9.18, I^2^ = 0%, P = 0.18) (Fig 3C). Diogenes et al. [30] showed that there was no association between infant TBLH-BA at 5 weeks postpartum and calcium and vitamin supplementation during pregnancy.

#### 3.4.4 Femur length (FL) and Humeral length (HL)

FL was examined in 3 RCTs [17, 30, 34] involving 333 participants, and HL was examined in 2 RCTs [17, 30] involving 186 participants. FL and HL assessments were performed on the offspring in utero and at birth. There was no association between vitamin D supplementation during pregnancy and FL in the offspring in the third trimester (MD 0.16, 95% CI -0.07, 0.40, I^2^ = 60%, P = 0.17) (Fi 3D). HL in the offspring during the third trimester of the experimental group was higher than that of the control group (MD 0.13, 95% CI 0.06, 0.21, I^2^ = 0, P = 0.0007) (Fig 3E). Vafaei et al. [17] showed that FL in the intervention group was not statistically significantly different during the first trimester compared with the controls but was significantly increased in the intervention group during the second and third trimesters. The HL of the intervention group was significantly higher than that of the control group in the second and third trimesters, but the difference was not statistically significant. Daniel et al. [34] showed that FL at birth did not significantly differ between infants born to mothers in the vitamin D vs. placebo groups.

### 3.5 Physical growth assessment

#### 3.5.1 Length

**Fig 4:**
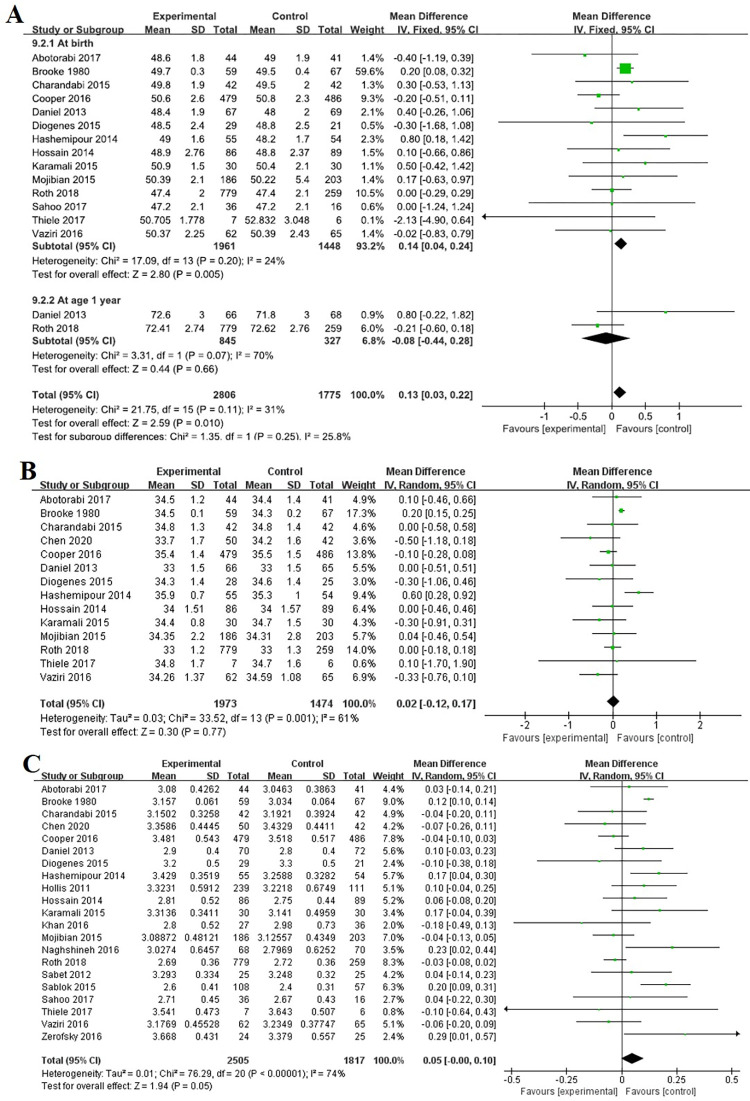
Forest plots of summary crude risk ratios of the association between vitamin D supplementation during pregnancy in the offspring physical growth assessment. Notes: A, Length; B, Birth head circumference; C, Birth weight.

Body length was assessed in 14 RCTs [18–23, 27, 29–34, 37] involving 3454 participants. All 14 RCTs measured the length of the offspring at birth, two measured it at 1 year, and two measured it at 4 weeks, 8 weeks, and 5 weeks. There was a significant difference in length at birth between vitamin D supplementation during pregnancy and controls (MD 0.14, 95% CI 0.04, 0.24, I^2^ = 24%, P = 0.005) but there was no significant difference at 1 year (MD -0.08, 95% CI -0.44, 0.28, I^2^ = 70%, P = 0.66) (Fig 4A). However, Vaziri et al. [23] and Diogenes et al. [30] reported that length measurements of infants at 4 weeks, 5 weeks and 8 weeks of postpartum were not significantly different between vitamin D supplements and placebo.

#### 3.5.2 Head circumference

Head circumference was assessed in 15 RCTs [15, 16, 18, 20–23, 27, 29–34, 37] involving 4369 participants. Thirteen RCTs measured the offspring at birth, among which 1 RCT also measured the offspring at 1 year after birth. One RCT [15] measured the offspring at 6 years. There was no association between vitamin D supplementation during pregnancy and head circumference at birth (MD 0.02, 95% CI -0.12, 0.17, I^2^ = 61%, P = 0.77) (Fig 4B). Brustad et al. [15] observed no differences in head circumference at age 6 between high-dose and standard-dose vitamin D supplements during pregnancy. Daniel et al. [34] and Vaziri et al. [23] also observed no association in head circumference at 4 weeks and 8 weeks postpartum and at 1 year between the vitamin D supplements and placebo groups.

#### 3.5.3 Weight

Offspring Weight was assessed in 21 RCTs [16, 18–37] with 4680 participants. Twenty-one randomized controlled trials weighed the offspring at birth, including three that also weighed the offspring at 4 weeks, 5 weeks, 8 weeks and 1 year after birth. There was no association between vitamin D supplementation during pregnancy and the birth weight of the offspring (MD 0.05, 95% CI -0.00, 0.10, I^2^ = 74%, P = 0.05) (Fig 4C). Vaziri et al. [23], Diogenes et al. [30] and Daniel et al. [34] also reported that weight at 4 weeks, 5 weeks, 8 weeks and 1 year postpartum was not significantly different between the vitamin D supplements and placebo groups.

### 3.6 Cord blood 25(OH)D concentration

**Fig 5:**
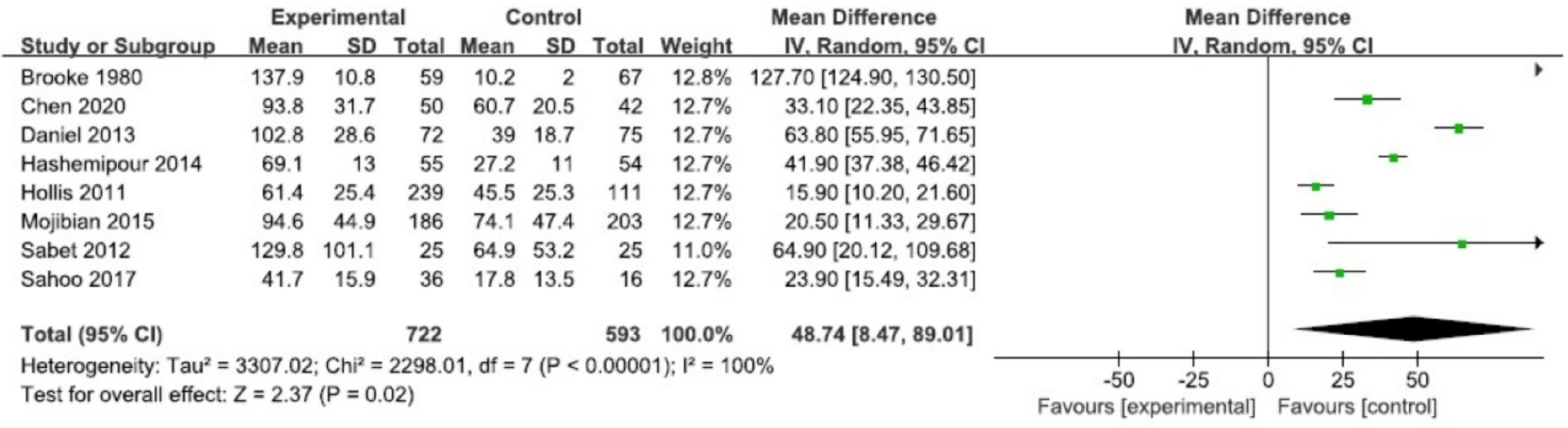
Forest plots of summary crude risk ratios of the association between vitamin D supplementation during pregnancy in the offspring cord blood 25(OH)D concentration.

Cord blood 25(OH)D concentration was assessed in 8 RCTs [16, 19, 29, 33–37] involving 1610 participants. Compared with the control group, the cord blood 25(OH)D concentration was significantly higher in the vitamin D intervention group (MD 48.74, 95% CI 8.47, 89.01, I^2^ = 100%, P = 0.02) (Fig 5). Considering that statistical heterogeneity exists among the study results (I^2^ = 100%), a sensitivity analysis was carried out by eliminating individual studies one by one. The results did not change directionally, suggesting that the meta-analysis results were relatively stable.

### 3.7 Subgroup analysis results

In addition, we used subgroup analysis to investigate the differential effects of the dose and the time of commencement of vitamin D on the offsprings’ physical growth assessments and cord blood 25(OH)D concentration. For daily, weekly and monthly dosages, we calculated the total amount in international units (IU) per day. The results of the meta-analysis are summarized in Table 2. For birth length, there was no significant difference between the control group and the vitamin D intervention 1001–2000 IU/day, 2001–3000 IU/day and 3001–4000 IU/day groups. However, compared with the control group, the birth length was significantly higher in the vitamin D intervention ≤1000 IU/day and ≥4001 IU/day groups (MD 0.14, 95% CI 0.03, 0.25, I^2^ = 35%, P = 0.01) (MD 0.61, 95% CI 0.16, 1.06, I^2^ = 0%, P = 0.008). Prenatal (third trimester) vitamin D supplementation was associated with a significant increase in birth length (MD 0.20, 95% CI 0.08, 0.32, I^2^ = 0%, P = 0.0009), while there was no significant difference if supplemented during the second trimester of pregnancy. For birth head circumference, no significant difference was observed between the control group and the different doses and initiation times of vitamin D interventions. Prenatal (second trimester) vitamin D supplementation was associated with a significant increase in birth weight (MD 0.07, 95% CI 0.00, 0.13, I^2^ = 66%, P = 0.04), while there was no significant difference for being supplemented during the first or third trimester of pregnancy. The cord blood 25(OH)D concentration was significantly higher in the vitamin D intervention groups than in the control group regardless of the dose and the time of commencement of vitamin D.

**Table 2:** Subgroup analysis results.

Subgroup	Number of studies	Results of heterogeneity test		Meta analysis results	
		P value	I^2^	MD (95% CI)	P value
**Dose (vitamin D supplementation in intervention group)**					
** (A) Birth length**					
** **≤1000 IU/day	5	0.19	35%	0.14 (0.03, 0.25)	0.01
** **1001–2000 IU/day	3	0.80	0%	-0.20 (-0.73, 0.33)	0.46
** **2001–3000 IU/day	2	1.00	0%	0.00 (-0.33, 0.33)	1.00
** **3001–4000 IU/day	5	0.65	0%	-0.04 (-0.33, 0.24)	0.77
** **≥4001 IU/day	2	0.38	0%	0.61 (0.16, 1.06)	0.008
** (B) Birth head circumference**					
** **≤1000 IU/day	5	0.009	70%	0.03 (-0.16, 0.22)	0.77
** **1001–2000 IU/day	2	0.23	30%	-0.15 (-0.57, 0.26)	0.47
** **2001–3000 IU/day	1	-	-	-0.00 (-0.21, 0.21)	1.00
** **3001–4000 IU/day	5	0.92	0%	-0.08 (-0.26, 0.09)	0.35
** **≥4001 IU/day	3	0.007	80%	0.09 (-0.55, 0.72)	0.79
** (C) Birth weight**					
** **≤1000 IU/day	7	<0.00001	85%	0.03 (-0.06, 0.12)	0.50
** **1001–2000 IU/day	5	0.19	35%	0.07 (-0.04, 0.18)	0.19
** **2001–3000 IU/day	2	1.00	0%	0.00 (-0.06, 0.06)	1.00
** **3001–4000 IU/day	7	0.28	19%	-0.01 (-0.07, 0.05)	0.69
** **≥4001 IU/day	3	0.10	57%	0.08 (-0.05, 0.21)	0.23
** (D) Cord blood 25(OH)D concentration**					
** **≤1000 IU/day	2	0.006	87%	100.44 (39.43, 161.45)	0.001
** **1001–2000 IU/day	2	0.79	0%	12.06 (6.66, 17.46)	< 0.0001
** **2001–3000 IU/day	1	-	-	30.00 (21.31, 38.69)	< 0.00001
** **3001–4000 IU/day	2	0.94	0%	20.76 (15.39, 26.13)	< 0.00001
** **≥4001 IU/day	3	< 0.00001	93%	46.47 (30.33, 62.62)	< 0.00001
**The time of commencement of vitamin D**					
** (A) Birth length**					
** **first trimester	0	-	-	-	-
** **second trimester	8	0.17	32%	0.01 (-0.17, 0.19)	0.90
** **third trimester	6	0.94	0%	0.20 (0.08, 0.32)	0.0009
** (B) Birth head circumference**					
** **first trimester	1	-	-	-0.50 (-1.18, 0.18)	0.15
** **second trimester	7	0.02	60%	0.06 (-0.14, 0.27)	0.54
** **third trimester	6	0.14	40%	0.01 (-0.23, 0.24)	0.94
** (C) Birth weight**					
** **first trimester	1	-	-	-0.07 (-0.26, 0.11)	0.42
** **second trimester	13	0.0004	66%	0.07 (0.00, 0.13)	0.04
** **third trimester	7	0.04	55%	0.04 (-0.04, 0.12)	0.36
** (D) Cord blood 25(OH)D concentration**					
** **first trimester	1	-	-	33.10 (22.35, 43.85)	< 0.00001
** **second trimester	4	< 0.00001	95%	25.72 (11.56, 39.87)	0.0004
** **third trimester	3	< 0.00001	99%	87.03 (34.32, 139.74)	0.001

## 4. Publication bias

**Fig 6:**
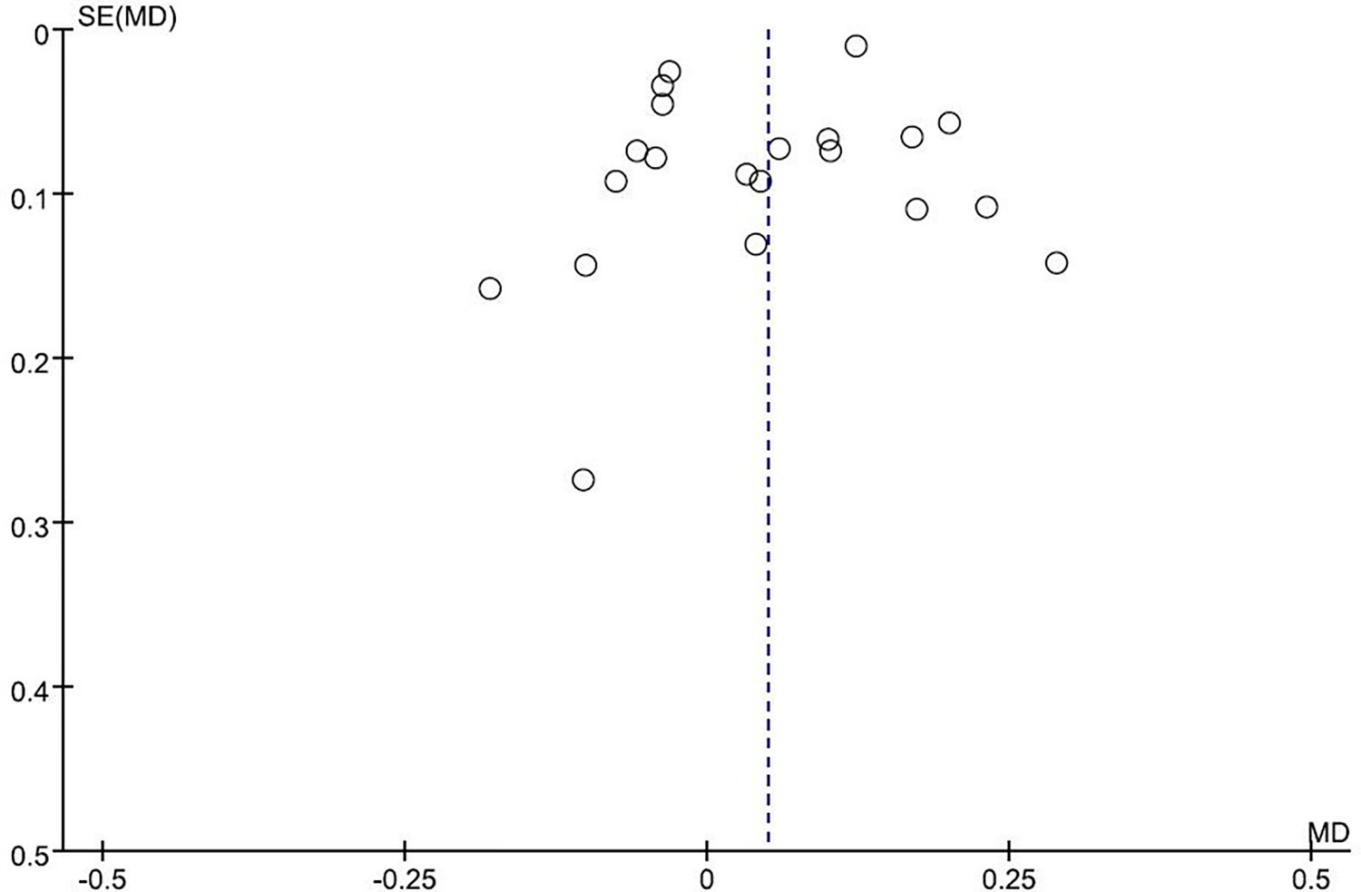
Funnel plot analysis depicting publication bias.

We assessed possible publication bias using a funnel plot. No obvious asymmetry was observed in the funnel plot (Fig 6).

## 5. Discussion

Vitamin D continues to attract substantial attention from clinicians, researchers, and the public. Dose recommendations differ among different organizations and countries. One study [38] proposed that vitamin D supplementation should be a mandatory basic care recommendation for all women, especially women of childbearing age and pregnant women. However, there remains a lack of consensus on target health outcomes, indications for prenatal supplementation, or evidence-based regimens for the supplement dose or strategies.

This is a systematic review and meta-analysis of the effects of vitamin D supplementation during pregnancy on bone development and offspring growth. The main finding of this systematic review and meta-analysis of RCTs was that vitamin D supplementation during pregnancy was associated with increased fetal HL in utero, increased body length at birth and a higher cord blood 25(OH) concentration. No association was observed between vitamin D supplementation and neonatal whole body BMC, neonatal whole body BMD, neonatal whole body BA, FL in utero (third trimester), length at 1 year, birth head circumference or birth weight. Different vitamin D doses and initiation times of supplementation had different results on neonatal health outcomes. Subgroup analysis revealed that vitamin D supplementation during pregnancy with both low and high doses (≤1000 IU/d and ≥4001 IU/d) was associated with a longer birth length. The time of commencement of vitamin D affected the birth length and birth weight results. Late vitamin D supplementation (during the second and third trimesters) improved birth weight and birth length, respectively. Evidence of an effect of vitamin D supplementation on long-term growth in children is lacking. Only a few studies have reported bone health and offspring growth at 1, 3, and 6 years of age. Therefore, the long-term results should be interpreted with caution, and we only carried out a descriptive analysis.

Vitamin D is derived from endogenous ultraviolet (UV)-induced synthesis and exogenous intake by supplementation. Due to the obvious seasonal variation in UV exposure, the serum 25(OH)D concentration also shows a similar variation pattern. In the absence of special interventions, the lowest circulating concentration of 25 (OH) D occurs in late winter or early spring [39, 40]. Hamgung et al. [41] observed that the total body bone mineral content (BMC) of newborns born in summer in South Korea was 8% higher than that of newborns born in winter, and the neonatal 25(OH)D concentration was positively correlated with the total BMC. The last trimester of pregnancy is a critical period for the accumulation of fetal bone mass, and maternal factors (such as obesity, physical activity, smoking, and 25(OH)D status) have a much stronger influence on the bone mineral content of offspring during the third trimester than in early pregnancy [42]. Therefore, we found that most of the included studies involved vitamin D supplementation during the second or last trimester of pregnancy. Adrian et al. [43] found that UV exposure in pregnant women during the third trimester of pregnancy was associated with trunk BMD, BMC and BA of offspring. Among the studies we included, 2 reported the importance of seasonal subgroups. Cooper [22] showed that the positive effects of prenatal vitamin D intervention on BMC, BMD and BA only existed in the subgroups born in winter, and such differences disappeared after the combination of seasonal subgroups. Brustad et al. [15] found that high-dose vitamin D supplementation during pregnancy had a sustained effect on the BMC and BMD of offspring, and they also found that the improvement was significantly better in the winter-born subgroup than in the other seasonal subgroups. The remaining studies did not specifically address the impact of season. Nine of the included studies [17, 20, 23, 24, 27, 29, 31, 33, 35] were conducted in Iran, where the clothing habits of local women expose them to insufficient ultraviolet radiation, and hinder the pathway of endogenous vitamin D synthesis, which may also affect the impacts of vitamin D supplementation.

The results of this systematic review suggested that vitamin D supplementation during pregnancy may not be associated with increased WB-BMC, WB-BMD and WB-BA in neonates. Several observational studies have shown an association between maternal vitamin D status during pregnancy and bone mineralization in offspring, but the results have been mixed. Weiler et al. [44] found that neonates with cord blood 25 (OH)D concentrations lower than 37.5 mol/L had lower BMCs. Viljakainen et al. [10] used the median 25 (OH) D concentration of the tested pregnant women as the grouping basis and found that a concentration above the median during pregnancy of 25 (OH)D resulted in a 13.9% higher tibial BMC and a 16.3% higher cross-sectional area compared with those below the median. However, tibia volumetric BMD did not differ between these groups. Follow-up of the same cohort [45] found that differences in tibial BMC had been eliminated at 14 months in the offspring, but differences in tibial cross-sectional area still existed. Other studies have also found opposite results. Prentice et al. [46] studied a population with 25 (OH)D concentrations exceeding 50 nmol/L, and the results showed that the vitamin D status of pregnant women during pregnancy was not correlated with the whole-body BMC and bone area of their offspring at 2, 13 and 52 weeks. Javaid et al. [12] showed that 25(OH)D in the third trimester of pregnancy was associated with the whole-body BMD, BMC and BA of offspring at the age of 9. In conclusion, the results of observational studies showed that vitamin D status during pregnancy might be positively correlated with offspring growth and development. However, due to differences in observation conclusions, further validation through randomized controlled trials is needed. The results of this systematic evaluation showed that prenatal vitamin D intervention could improve the skeletal measurement effect of offspring, but its effect on bone mass accumulation is not ideal. These null effects may be due to the small sample size included in the trials. These conclusions suggest that we should rethink the relationship between vitamin D supplementation during pregnancy and fetal bone mineralization.

Anthropometry outcomes were most commonly reported among the included trials. The results of this systematic review suggested that prenatal vitamin D supplementation may be associated with longer body length at birth. There was no association between vitamin D supplementation during pregnancy and birth weight or birth head circumference. To the best of our knowledge, several systematic reviews on the effects of vitamin D intervention during pregnancy on offspring have been published, but the results are controversial. Roth de et al. [47] indicated that prenatal vitamin D supplementation increased mean birth weight, and increased infant length at 1 year of age. There was a lack of evidence of prenatal vitamin D supplementation on birth length or head circumference. Only generally healthy pregnant women were included in our study, while Roth de et al. [47] also included pregnant women with gestational diabetes, hypocalcemia and multiple sclerosis. Therefore, the studies included in the analysis were different. A 2019 update of the Cochrane Collaboration systematic review and meta-analysis of trials of vitamin D in pregnancy (versus placebo or no supplement) conducted by Palacios C et al. [48] suggested a longer birth length among infants from women taking vitamin D supplementation during pregnancy compared to women in the no treatment or placebo group, while it probably made little or no difference in head circumference and weight at birth compared to no treatment or placebo. The consistency of the systematic evaluation conclusions may be limited by the following reasons: the measurement methods used in the original study are different, and the measurement results have systematic errors. The absolute value of the physical measurement was small, the accuracy was low, and the difference in measurement results could not be fully displayed. Because of the different inclusion and exclusion criteria of the systematic review, there were differences in the selection of documents. Different grouping policies were used when data are merged.

The subgroup analysis results of this study showed that there was no significant difference between the control group and the 1001–4000 IU/day vitamin D intervention groups. However, compared with the control group, the birth length was significantly higher in the vitamin D intervention ≤1000 IU/day and ≥4001 IU/day groups. High levels of vitamin D supplementation (>10,000 IU/d or 250 Wg/d) may lead to hypervitaminosis, hypercalcemia and hypercalciuria [48]. Vitamin D supplementation at 4000 IU/d is the upper limit established by the Institute of Medicine in the USA [49]. Of all the included studies, the vitamin intervention doses in only three studies were more than 4000 IU/d [16, 33, 34]. A previous systematic review and meta-analysis showed that low-dose vitamin D supplementation (≤2000 IU/d) was associated with a reduced risk of fetal or neonatal mortality, but higher doses (>2000 IU/d) did not reduce this risk [3]. Due to the limitations of the number of studies, the assessment of the safety of high-dose vitamin D supplementation (more than 4000 IU/d) compared with placebo or regular dose is lacking. Therefore, for healthy pregnant women, high-dose supplementation still needs to be conducted with caution. Additionally, prenatal (third trimester) vitamin D supplementation was associated with a significant increase in birth length, and prenatal (second trimester) vitamin D supplementation was associated with a significant increase in birth weight. These results indicated that late vitamin D supplementation (during the second and third trimesters) may have a greater impact on the growth of the offspring. These different subgroup analysis results suggested that in future vitamin D supplementation strategies, different supplement doses and initiation times should be considered.

We also found that vitamin D supplementation during pregnancy increased cord blood 25(OH)D concentrations. The results remained stable regardless of the dose and the time of commencement of vitamin D. There was large heterogeneity in these results, which could be related to the differences in vitamin D doses and measuring methods. Maternal vitamin D status is important as the fetus completely relies on this source. The increase in blood 25(OH)D concentration may explain the potential neonatal health outcomes.Several cohort studies [50, 51] observed a significant inverse correlation between cord blood 25(OH)D concentration and birthweight. In contrast, a cohort study conducted in Australia found that standardized 25(OH)D in cord blood was not associated with length, weight or head circumference at birth, 18 months or 4 years of age [52]. Because these results are still controversial, replication of these results will be needed in larger study populations.

## 6. Limitations

Several limitations of the study should be acknowledged. First, due to the limited number of original studies and the scattered time points at which outcomes were measured, many results could not be combined. Second, outcome measurements were quite different across individual studies; therefore, for each outcome, there were only a few RCTs, and in the combined studies, pregnant women differed significantly in baseline status, vitamin D supplementation dose, and supplementation starting point, which may have diluted the significance of certain specific results. Third, most studies had small sample sizes, which may not be sufficient to test the actual effects of vitamin D interventions during pregnancy.

## 7. Conclusions

A systematic review of randomized clinical trials suggests that vitamin D supplementation during pregnancy may be associated with increased humeral length (HL) in utero, increased body length at birth and a higher cord blood 25(OH) D concentration. Supplementation starting in the second or third trimester had a greater influence. No effect of vitamin D supplementation during pregnancy on bone health in offspring was observed because of the limited number of studies. Evidence of the effect of supplementation on long-term growth in children is lacking. The current evidence is not sufficient to guide vitamin D supplementation strategies for pregnant women, but the results suggest that we should pay more attention to the benefits in different regions and seasons, and the doses and initiation times of supplementation. Additional rigorous high quality, long-term and larger randomized trials are required in the future to evaluate different vitamin D supplementation regimens in pregnancy to assess their effects on bone development and growth in children. Different vitamin D doses, different frequencies, different forms, different types, different combinations of medications and different commencement periods should be considered.

## Supporting information

S1 File Forest plots of summary crude risk ratios of sub-group meta-analyses. (DOCX)

S2 File PRISMA checklist. (DOCX)

S3 File Search strategy for electronic databases. (DOCX)
