# Supplementary material for: Effects of vitamin D supplementation during pregnancy on bone health and offspring growth: A systematic review and meta-analysis of randomized controlled trials
Source: PLoS One. 2022 Oct 13;17(10):e0276016. doi: 10.1371/journal.pone.0276016 (PMC9560143; doi:10.1371/journal.pone.0276016)
Supplement: S1 File — (DOCX) [file pone.0276016.s001.docx]

S1 File: Forest plots of summary crude risk ratios of sub-group meta-analyses.

(A) Birth length


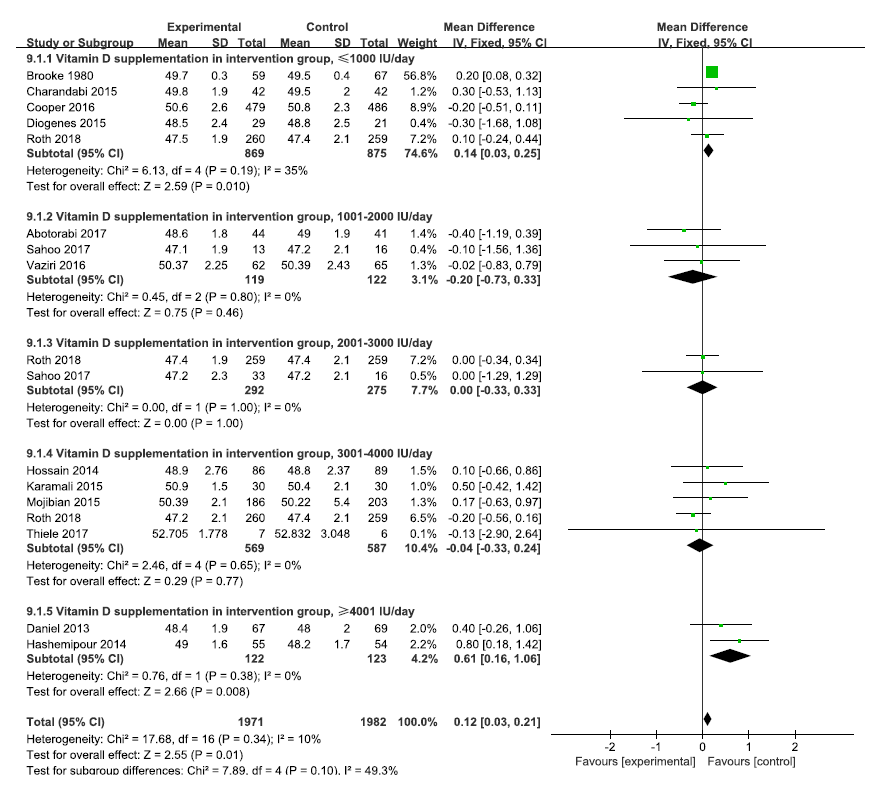


(B) Birth head circumference


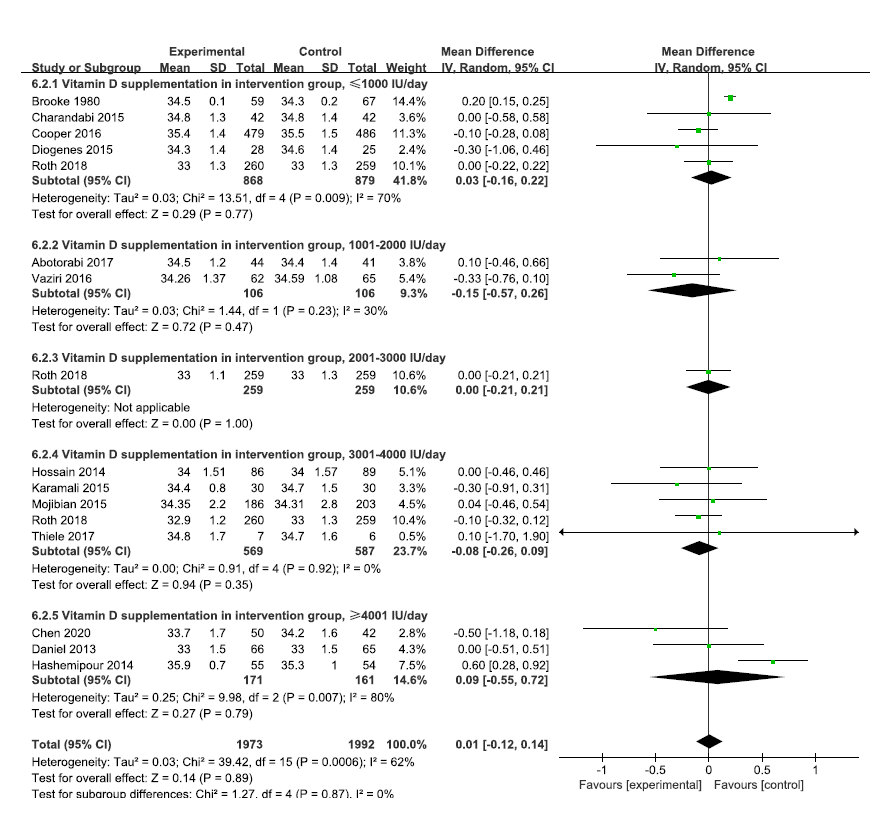


(C) Birth weight


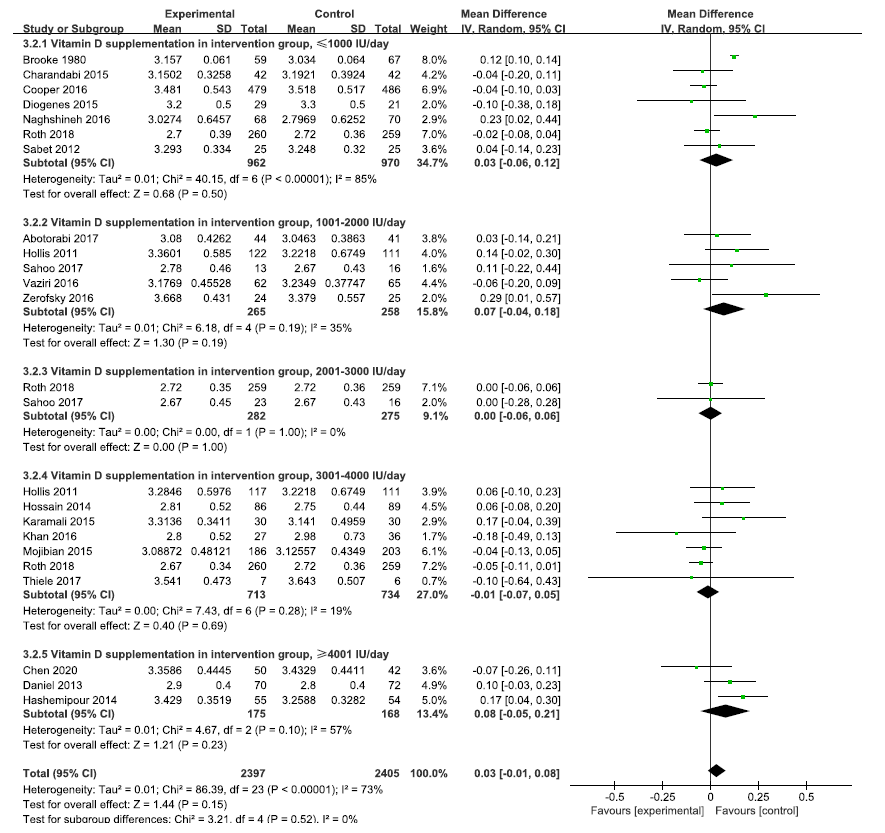


**Fig S1. subgroup analysis of the association between different doses of vitamin D supplementation during pregnancy in the offspring physical growth assessment.**

1. Birth length


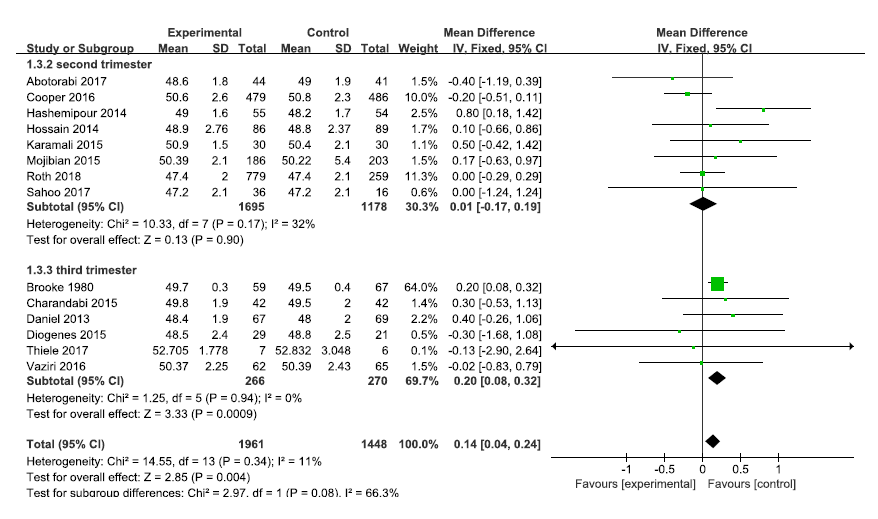


1. Birth head circumference


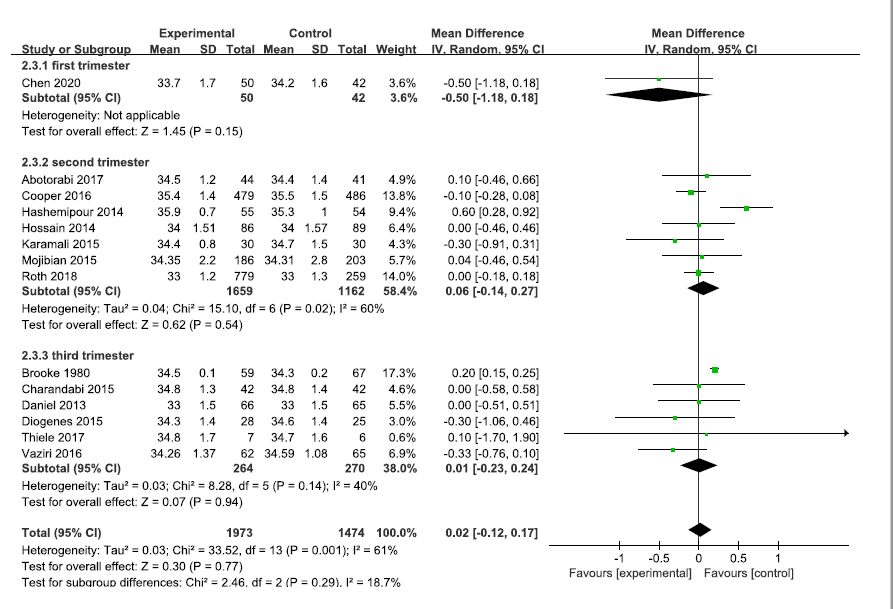


(C) Birth weight


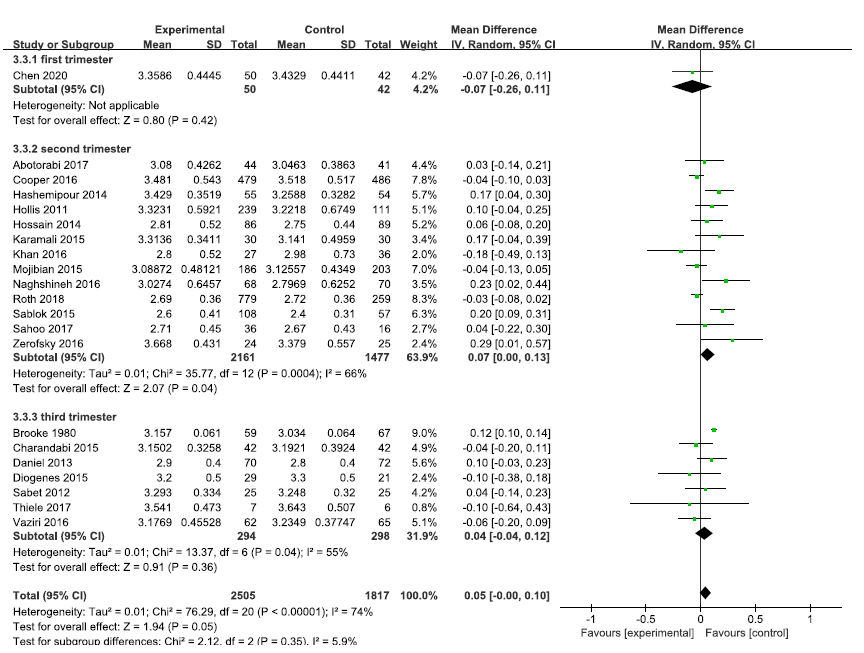


**Fig S2. subgroup analysis of the association between the initiation time of vitamin D supplementation during pregnancy in the offspring physical growth assessment.**


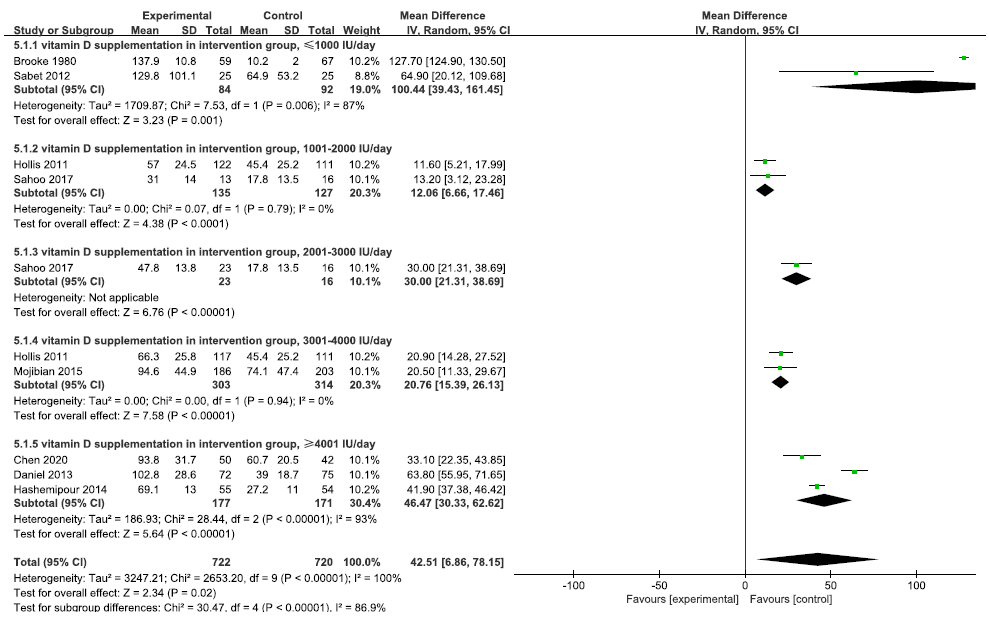


**Fig S3. subgroup analysis of the association between different doses of vitamin D supplementation during pregnancy in cord blood 25(OH)D concentration.**


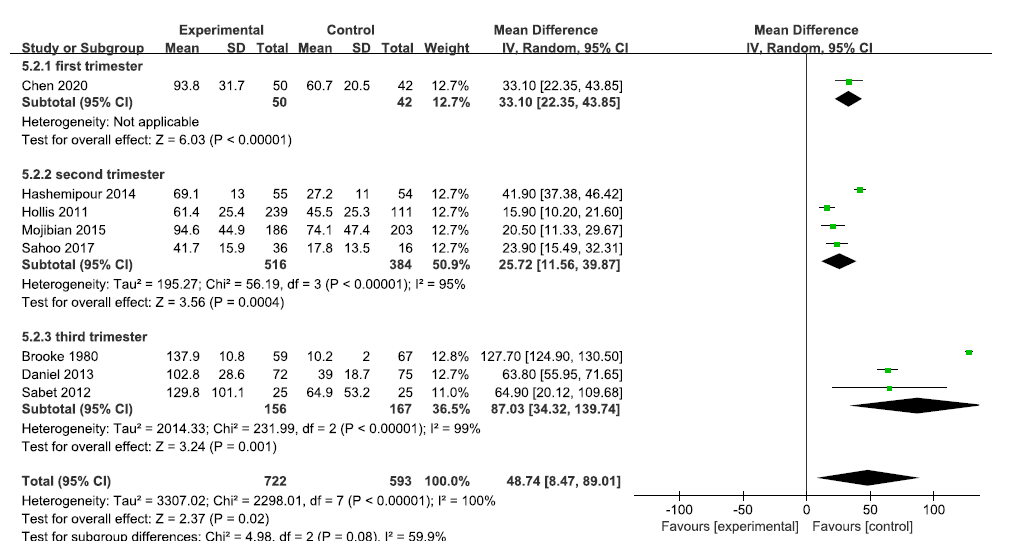


**Fig S4. subgroup analysis of the association between the initiation time of vitamin D supplementation during pregnancy in cord blood 25(OH)D concentration.**
