# Supplementary material for: Effects of vitamin D supplementation during pregnancy on bone health and offspring growth: A systematic review and meta-analysis of randomized controlled trials
Source: PLoS One. 2022 Oct 13;17(10):e0276016. doi: 10.1371/journal.pone.0276016 (PMC9560143; doi:10.1371/journal.pone.0276016)
Supplement: S3 File — (DOCX) [file pone.0276016.s003.docx]

**S3 File. Search strategy for electronic databases.**

| Search | Query |
| --- | --- |
| [#4](https://www-ncbi-nlm-nih-gov.elibrary.einstein.yu.edu/pubmed/advanced) | #1 AND #2 AND #3 |
| [#3](https://www-ncbi-nlm-nih-gov.elibrary.einstein.yu.edu/pubmed/advanced) | “offspring*”[Title/Abstract] OR "infan*"[Title/Abstract] OR "perinatal"[Title/Abstract] OR "neonatal"[Title/Abstract] OR "early life"[Title/Abstract] OR "child*"[Title/Abstract] OR "adolescen*"[Title/Abstract] OR "adult*"[Title/Abstract] OR "fetus"[Title/Abstract] |
| [#2](https://www-ncbi-nlm-nih-gov.elibrary.einstein.yu.edu/pubmed/advanced) | "Vitamin D"[Title/Abstract] OR "Vit D"[Title/Abstract] OR "25(OH)D"[Title/Abstract] OR "25-hydroxyvitamin D"[Title/Abstract] OR "25OH-vitamin D"[Title/Abstract] |
| [#1](https://www-ncbi-nlm-nih-gov.elibrary.einstein.yu.edu/pubmed/advanced) | “Maternal”[Title/Abstract] OR "prenatal" [Title/Abstract] OR "cord"[Title/Abstract] OR "in utero"[Title/Abstract] OR "pregnan*"[Title/Abstract] OR "mother"[Title/Abstract] OR "gestation*"[Title/Abstract] OR "antenatal"[Title/Abstract] OR "perinata"[Title/Abstract] |
